# Managing Bilateral Vernal Keratoconjunctivitis, Keratoconus, and Steroid-Induced Glaucoma: A Threefold Struggle

**DOI:** 10.7759/cureus.77901

**Published:** 2025-01-24

**Authors:** Nur Hafizah Maffar, Yaakub Azhany, Nur Ain Shafiyah Mohd Ghazali, Julieana Muhammed

**Affiliations:** 1 Department of Ophthalmology and Visual Science, School of Medical Sciences, Universiti Sains Malaysia, Kubang Kerian, MYS

**Keywords:** corticosteriod, flare-up, keratoconus (kc), steroid-induced glaucoma, vernal keratoconjunctivitis

## Abstract

The coexistence of bilateral vernal keratoconjunctivitis (VKC), keratoconus, and steroid-induced glaucoma presents a complex ocular challenge, threatening visual acuity and long-term eye health. This combination poses significant risks, with VKC and keratoconus progressively affecting both eyes while glaucoma, induced by necessary steroid treatments, further complicates the clinical picture. We report a case of a young Malay girl who complained of bilateral eye itchiness with progressive blurring of vision, a history of frequent changes in prescribed glasses, and vigorous eye rubbing. Diagnosed with VKC at age 14, she defaulted on follow-up and began unsupervised use of topical steroids during flare-ups. This resulted in steroid-induced glaucoma, complicating the management of both VKC and coexisting keratoconus. This case underscores the importance of careful, supervised treatment, as improper management can significantly affect long-term outcomes.

## Introduction

Vernal keratoconjunctivitis (VKC) is a chronic allergic conjunctivitis characterized by intense itching, photophobia, tearing, and mucus discharge. Its hallmark signs include giant papillae on the upper tarsal conjunctiva and limbal inflammation with Trantas dots [1,2]. VKC predominantly affects young men and tends to worsen seasonally, correlating with environmental allergen exposure [3,4]. Although VKC generally resolves after adolescence, its chronic nature can lead to secondary complications such as keratoconus and glaucoma, particularly when corticosteroids are used long-term. The incidence of glaucoma in patients with VKC receiving corticosteroid therapy ranges from 2% to 7% [5,6].

Keratoconus is a progressive corneal ectatic disorder characterized by paracentral corneal thinning and protrusion, resulting in an irregular corneal shape that impairs vision. It commonly begins during adolescence, coinciding with the peak incidence of VKC. Vigorous eye rubbing, frequently seen in VKC patients, is a significant risk factor for keratoconus, exacerbating biomechanical corneal changes [1,4]. Keratoconus causes blurred vision, light sensitivity, myopia, and irregular astigmatism, and if untreated, can lead to severe complications such as corneal scarring and acute hydrops [1].

Topical corticosteroids, commonly used to manage VKC symptoms, provide rapid and effective relief, earning them the nickname "magic drug". However, corticosteroids carry the risk of steroid-induced glaucoma, particularly in children and adolescents [7,8]. This population is more susceptible to elevated intraocular pressure (IOP), which can lead to optic nerve damage and permanent visual field loss if not carefully monitored. The risk of steroid-induced glaucoma increases with prolonged use of potent corticosteroids, especially when used without supervision [6].

In this case report, we present a young Malay girl with VKC complicated by progressive keratoconus and steroid-induced glaucoma, illustrating the complex clinical challenges of managing these interconnected conditions.

## Case presentation

An 18-year-old Malay girl with a history of allergic rhinitis and bronchial asthma, who uses Nasonex nasal spray OD, MDI Budesonide BD, and MDI Salbutamol PRN, presented with bilateral eye itchiness and progressive blurring of vision. She reported frequent changes in her eyeglass prescription and a history of vigorous eye rubbing. Her symptoms were consistent with VKC, which had been diagnosed five years prior; however, she had defaulted on regular follow-up appointments. Due to frequent VKC flare-ups, she had resorted to using over-the-counter topical steroids without ophthalmologist supervision.

At presentation, her best-corrected visual acuity (BCVA) was 6/36 in the right eye and 6/9 in the left eye. Examination revealed macropapillae on the upper tarsal conjunctiva and limbitis with Trantas dots. Refraction showed significant myopia with irregular astigmatism (right eye: -5.25DS / -3.25DC x 175°, left eye: -4.75DS / -3.00DC x 155°), suggestive of keratoconus. The patient's prescription had been changing frequently, and she had increasing difficulty achieving clear vision with standard corrective lenses.

Computerized corneal topography was performed using the Carl Zeiss Meditec Atlas 993 Corneal Topographer version A12.2 (ZEISS, Oberkochen, Germany), confirming the diagnosis of keratoconus. The results showed marked asymmetric bow-tie astigmatism, with a skewed radial axis, corneal steepening, thinning, and irregularity. This prompted the decision to proceed with bilateral corneal cross-linking to halt disease progression. Despite undergoing the cross-linking procedure, her vision worsened, and she developed increased sensitivity to light. Several VKC flare-ups ensued, prompting her to resume unsupervised use of topical steroids for inflammation control.

**Figure 1:**
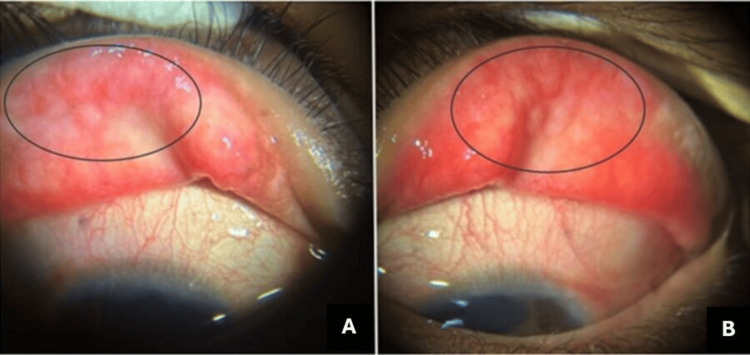
Macropapillae appearance on the upper tarsal conjunctiva (highlighted with the circle), with A representing the right eye and B representing the left eye

**Figure 2:**
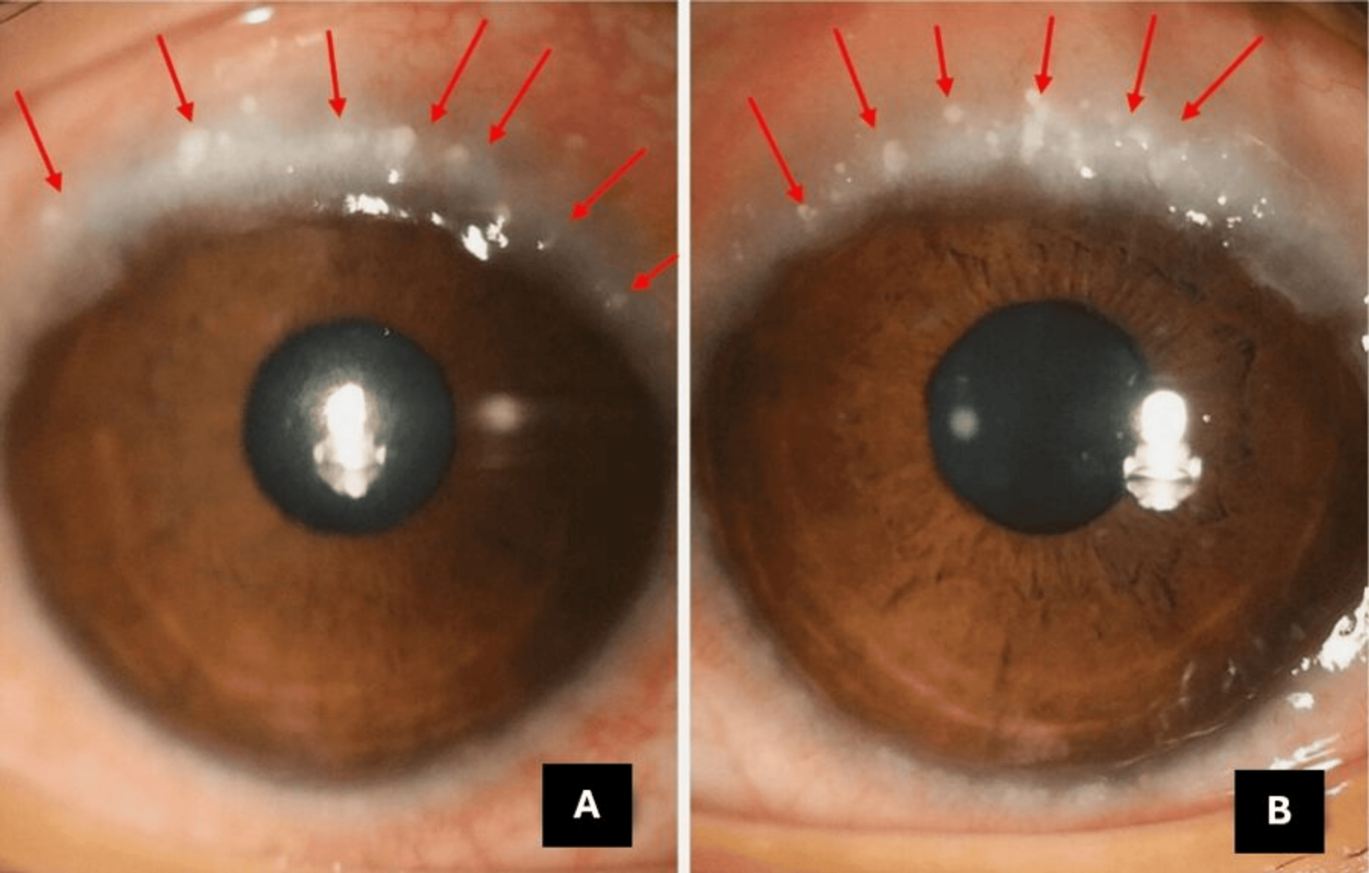
The anterior segment displays 360º chronic severe limbitis and Trantas dots in both eyes. The right eye is labeled as A, and the left eye as B, with red arrows indicating the findings

**Figure 3:**
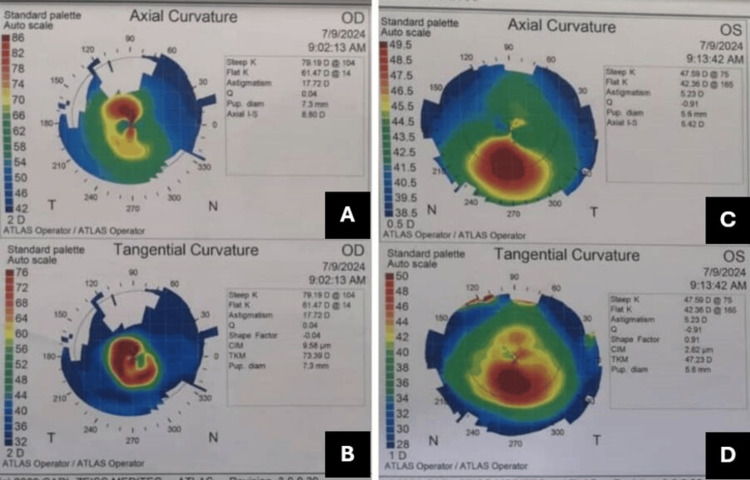
Corneal topography following corneal cross-linking reveals advanced keratoconus progression in the right eye (A and B) and minimal progression in the left eye (C and D)

At a follow-up visit, her visual acuity had deteriorated to hand movements in the right eye, while the left eye maintained a BCVA of 6/9. Examination revealed macropapillae on the upper tarsal conjunctiva (Figure 1), chronic severe limbitis with Trantas dots (Figure 2), and marked progression of keratoconus with thin corneal thickness in the right eye and minimal progression of keratoconus in the left eye (using Carl Zeiss Meditec Atlas 9000 Corneal Topographer revision 3.0) (Figure 3). For the right eye, the pachymetry was 390μm, with a mean K of 69.90D, simulated K of 17.72D, and axial I-S of 8.80D. In the left eye, the pachymetry was 469μm, with a mean K of 44.98D, simulated K of 5.23D, and axial I-S of 5.42D.

These findings highlight significant progression in keratoconus in the right eye, as evidenced by the reduced corneal thickness and elevated keratometry readings, which indicate severe corneal ectasia. This progression calls for closer monitoring and potential adjustments to the management plan to prevent further visual deterioration. In contrast, the left eye shows minimal progression, suggesting better stability, though continued observation is necessary. The presence of macropapillae and limbitis with Trantas dots indicates active inflammation, which may exacerbate the keratoconus and impact long-term visual outcomes. These findings are crucial for tailoring the management strategy, which may include anti-inflammatory treatment, careful monitoring, and consideration of additional therapeutic interventions as needed.

**Figure 4:**
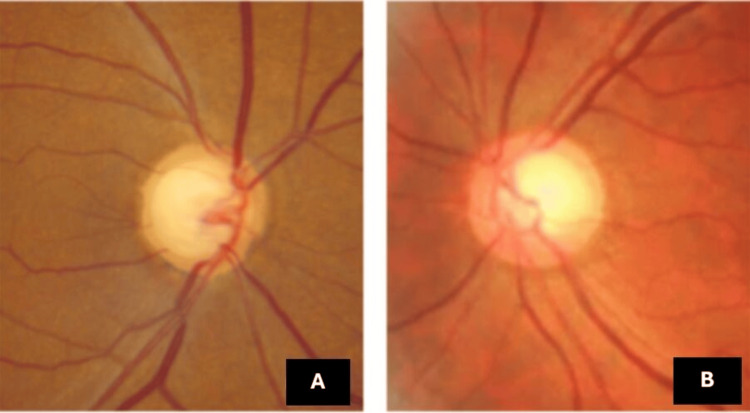
Fundus photographs show an advanced cup-to-disc ratio of 0.95 with pallor in the right eye (A) and a ratio of 0.6 with a pink appearance in the left eye (B)

IOP was markedly elevated at 54 mmHg and 20 mmHg in the right and left eye, respectively. Fundus examination showed an advanced optic disc cupping of 0.95 pale in the right eye and 0.6 pink in the left eye (Figure 4), indicating significant optic nerve damage.

**Figure 5:**
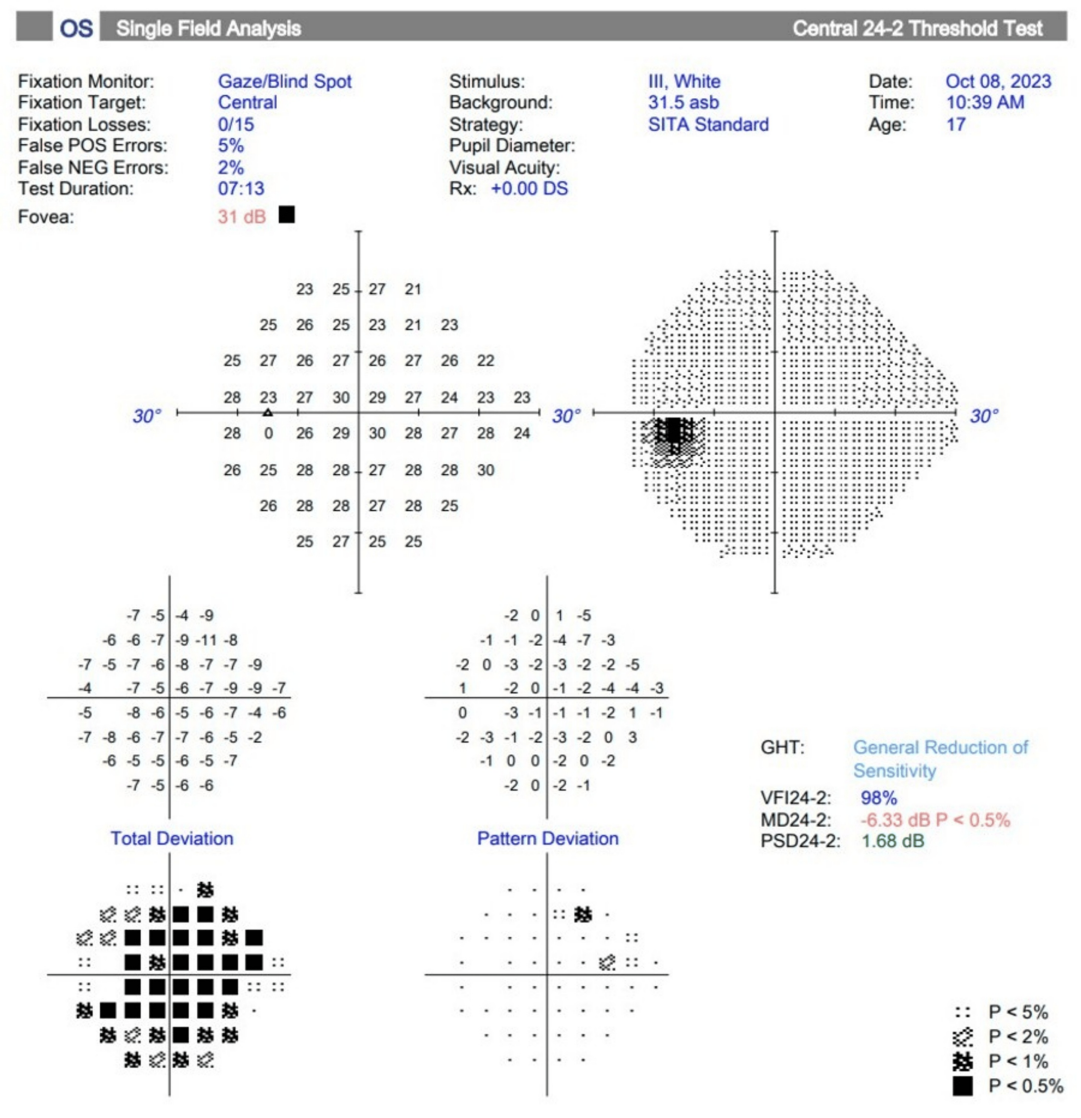
HVF test of the left eye (OS) is within normal limits. Unable to perform the HVF test of the right eye (OD) due to severely reduced vision, limited to hand movement perception HVF: Humphrey visual field

The Humphrey visual field test for the right eye could not be performed due to severely reduced vision, limited to hand movement perception. However, the visual field results for the left eye (Figure 5) show that the glaucoma hemifield test (GHT) indicates a generalized reduction in sensitivity, while the pattern standard deviation (PSD) remains normal. These findings reflect the diffuse effects of elevated IOP in the early stages of glaucoma. The generalized reduction seen in the GHT suggests uniform retinal sensitivity loss caused by global ischemic or mechanical stress on retinal ganglion cells. This diffuse effect is typical of early stages of glaucoma and does not involve the focal nerve damage characteristic of advanced glaucoma. The PSD, which measures variability in sensitivity across the visual field, remains normal because no significant localized defects, such as arcuate scotomas or nasal steps, are present. This pattern suggests early-stage disease, where timely IOP reduction can prevent further damage.

**Figure 6:**
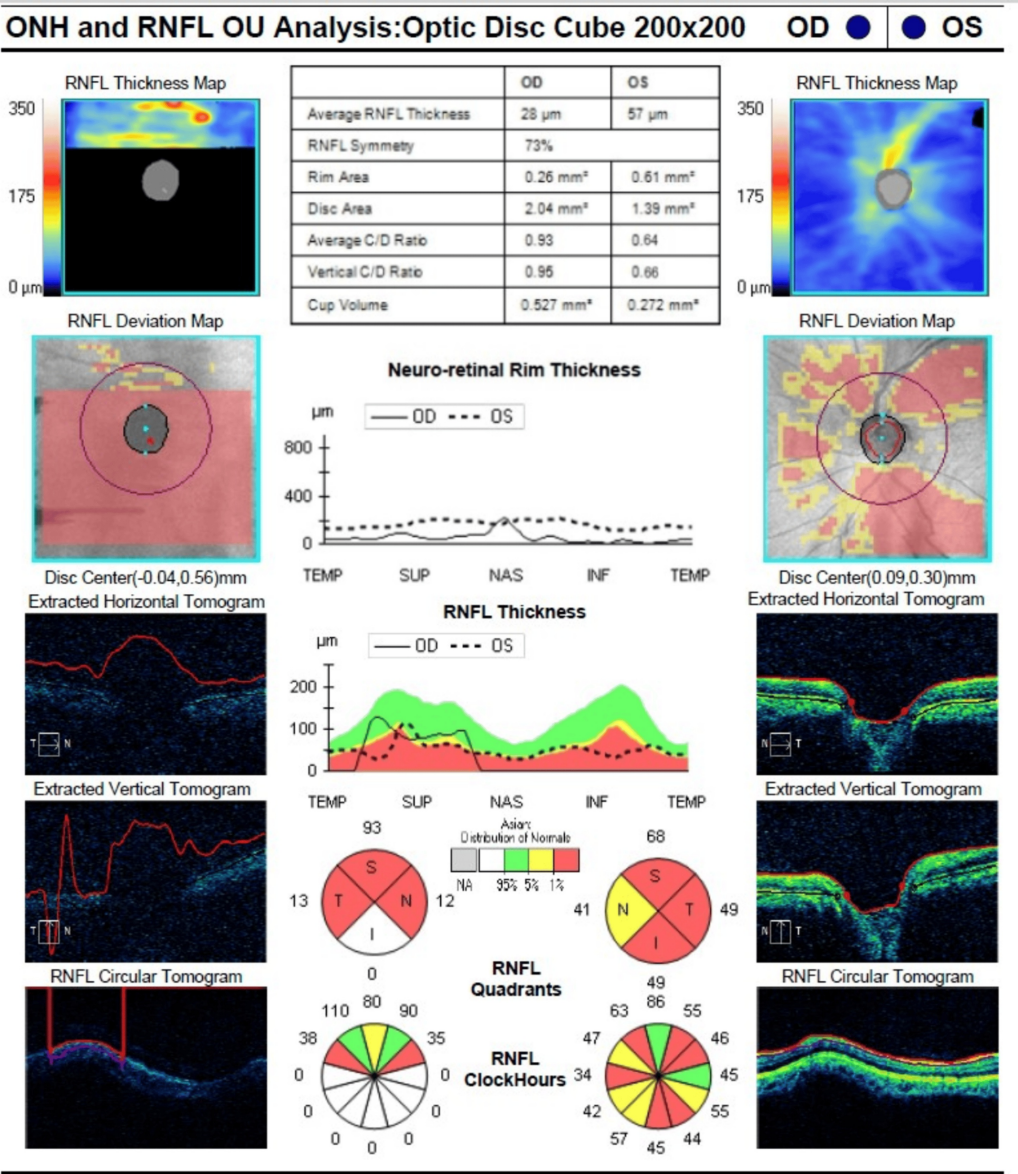
OCT RNFL showed thinning of both nerve fiber layers, more pronounced in the right eye (OD) OCT: Optical coherence tomography; RNFL: retinal nerve fiber layer

Optical coherence tomography (OCT) of the retinal nerve fiber layer showed thinning in both eyes, with more pronounced thinning in the right eye (Figure 6).

A diagnosis of steroid-induced glaucoma was made, attributable to the prolonged and unsupervised use of topical steroids following cross-linking. To date, the patient’s IOP is well-controlled with two anti-glaucoma medications and no surgical intervention.

## Discussion

Topical steroids are crucial for managing various ocular conditions due to their potent anti-inflammatory effects. However, injudicious use and insufficient monitoring can lead to complications like ocular hypertension, glaucoma, and cataracts, significantly affecting vision. While cataract-related vision loss is often reversible, glaucoma, which can progress unnoticed without early intervention, can result in permanent visual impairment [5-9]. This risk is particularly high in patients with VKC, a chronic allergic conjunctival inflammation common in young individuals in tropical climates [1,3-6]. VKC is driven by Type I and IV hypersensitivity reactions, where T-cell stimulation leads to the release of cytokines that activate eosinophils and mast cells, causing intense itching and burning, especially during the summer months [1,3,4].

Approximately 85% of VKC patients may require corticosteroid therapy during their illness [1-4]. While corticosteroids effectively control VKC symptoms, they can also elevate IOP by altering the trabecular meshwork (TM), reducing aqueous humor outflow [10,11]. This disruption in the TM cytoskeleton and morphology, triggered by increased extracellular matrix deposition and activation of the myocilin gene, leads to steroid-induced glaucoma, affecting between 2% and 7% of VKC patients [5,6,12,13].

The risk of steroid-induced glaucoma varies with the type and duration of corticosteroid use. Dexamethasone has a higher risk of inducing glaucoma compared to prednisolone or fluorometholone, although even the latter can cause elevated IOP with prolonged use. Elevated IOP can develop within hours to weeks after starting treatment, and long-term use beyond 8-10 weeks can result in sustained high IOP, even after discontinuation. This suggests that long-term steroid use can lead to permanent changes in the TM’s microstructure [10,11].

In addition to topical corticosteroids, asthma and rhinitis medications, particularly inhaled corticosteroids (ICS) and intranasal corticosteroids (INS), may contribute to the observed ocular changes. These medications, while effective in controlling inflammation, can also exacerbate the risk of posterior subcapsular cataract, elevated IOP, and steroid-induced glaucoma, further complicating the management of VKC. According to Vinokurtseva et al., an overall analysis revealed a statistically significant increase of 0.69 mmHg in IOP among users of ICS or INS compared to non-users [14].

The increasing prevalence of allergic eye diseases, particularly in children under 10 who are more susceptible to rapid IOP increases, underscores the need for careful steroid use in pediatric patients. Regular follow-up and stringent monitoring are critical in preventing serious outcomes [5,6,8,12]. A multidisciplinary approach involving ophthalmologists and allergists, combined with thorough patient education, is essential to minimize the risks associated with steroid therapy and ensure optimal long-term visual health.

To reduce reliance on long-term corticosteroid use, newer therapeutic approaches such as calcineurin inhibitors (e.g., cyclosporine and tacrolimus) have gained attention. These alternatives are effective in controlling ocular inflammation with a lower risk of IOP elevation and glaucoma. Immunomodulatory therapies like cyclosporine A have shown promise in managing VKC symptoms over the long term without the same level of adverse effects associated with steroids [1,2,4]. For some patients, integrating non-steroidal anti-inflammatory drugs can also help control inflammation while minimizing the need for potent steroids.

However, it is important to note that calcineurin inhibitors, while effective, carry potential risks such as nephrotoxicity, hypertension, elevated blood lipids, and an increased risk of malignancies such as squamous cell carcinoma due to their immunosuppressive action. These side effects necessitate careful monitoring and individualized dosing to avoid long-term complications. Nephrotoxicity remains a significant concern, and careful evaluation of kidney function is required through regular blood creatinine levels. Additionally, hypertension and lipid abnormalities need to be monitored to prevent cardiovascular complications. The immunosuppressive nature of calcineurin inhibitors also increases the risk of malignancies, particularly squamous cell carcinoma, which demands cautious use and patient education on early signs of skin changes or abnormal growths.

In managing this case, several strategies have been considered to mitigate the risks associated with calcineurin inhibitors. Regular follow-up visits with both ophthalmologists and allergists are essential to monitor the patient’s response to treatment, including regular assessment of IOP, and kidney function through blood creatinine levels, blood pressure, and lipid profile. Individualized dosing of calcineurin inhibitors is crucial, starting at lower doses and gradually increasing to balance therapeutic efficacy while minimizing side effects. Additionally, combination therapies involving mast cell stabilizers, H1-antihistamines, and anti-inflammatory agents can help reduce allergic reactions without solely relying on calcineurin inhibitors [15,16]. Lifestyle modifications, such as using air purifiers and wearing protective eyewear, can also lessen flare-ups and the need for aggressive treatment. Other immunomodulatory agents like dupilumab (interleukin antagonist), which targets the IL-4/IL-13 pathway, have shown efficacy in managing allergic inflammation without the same risk profile as calcineurin inhibitors. Punctal occlusion can provide additional relief by reducing tear drainage and maintaining moisture, which helps soothe ocular irritation and prevent excessive eye rubbing [15,16].

By adopting a multifaceted approach that includes close monitoring, lifestyle modifications, and alternative therapies, VKC symptoms can be effectively managed while mitigating the risks associated with calcineurin inhibitors. Regular follow-ups are particularly essential for pediatric and high-risk patients, alongside pharmacological advancements.

Monitoring IOP during and after corticosteroid treatment is critical to prevent complications such as glaucoma and optic nerve damage. Educating patients and caregivers on the importance of adhering to scheduled eye exams, recognizing early signs of elevated IOP, and managing allergic triggers are fundamental to ensuring long-term care and improved outcomes.

Innovations in drug delivery systems, such as slow-release steroid implants and targeted topical formulations, are under development to reduce systemic absorption and minimize local complications. These advancements aim to provide sustained therapeutic effects while lowering the risks of steroid-induced side effects [17].

Ultimately, the long-term management of VKC and associated ocular conditions relies on vigilant monitoring, the integration of alternative therapies, and patient education. As these alternative therapies and delivery mechanisms continue to evolve, they offer hope for better management of chronic ocular conditions while protecting patients from the serious risks posed by prolonged steroid use.

## Conclusions

Managing VKC often involves a combination of avoiding potential allergens, modifying behavior, and adhering to complex treatment regimens using multiple topical and systemic agents. This case underscores the challenges in managing VKC alongside keratoconus, highlighting the detrimental consequences of unsupervised steroid use. While corneal cross-linking offers stabilization for keratoconus, indiscriminate over-the-counter steroid use for VKC resulted in steroid-induced glaucoma, optic nerve damage, and vision loss. Regular follow-ups and IOP monitoring are essential to mitigate such risks. Collaboration among ophthalmologists, optometrists, allergists, and primary care providers is crucial for optimizing VKC management and minimizing long-term steroid-related complications, thereby preserving vision and improving quality of life.
